# How Power Influences Decision-Makers’ Investment Behavior in the Domains of Loss and Gain

**DOI:** 10.3390/ijerph182312834

**Published:** 2021-12-06

**Authors:** Katarzyna Sekścińska, Joanna Rudzinska-Wojciechowska

**Affiliations:** 1 Faculty of Psychology, University of Warsaw, 00-183 Warsaw, Poland; 2 Center for Economic Psychology and Decision Sciences, Kozminski University, 03-301 Warsaw, Poland; jrudzinska@kozminski.edu.pl

**Keywords:** power, investment decisions, gain, loss

## Abstract

We present a study (*N* = 645) investigating how power alters people’s propensity to take investment risks in a changing decision context of gains and losses and the intensity of their reactions to this experience. The results indicate that people in a state of power made more risky investment decisions than the control group regardless of prior gain or loss outcome, whereas people lacking power took less investment risk than the control group, regardless of previous outcomes. Moreover, people with power and those lacking power differed in their reactions to gains and losses, with the former reacting more to gains and the latter to losses.

## 1. Introduction

Individual investors experience financial gains and losses on a daily basis. Although neoclassical economics suggests that they should make each decision independently of previous experiences, based only on expected value or utility [1], undoubtedly, past decisions and their outcomes impact current choices. It is well documented that previous gains and losses impact consecutive risky financial choices (for example: [2,3]). However, the nature of this influence is not entirely understood. Some studies have found that people become more risk-seeking after experiencing success [2,4,5], but others show that risk appetite is greater after experiencing losses [2,6]. We propose that people make different investment decisions after the same experience (e.g., a gain or loss) depending on whether they possess or lack power. Our proposal is based on a vast body of research indicating that power changes the way people react to successes and failures and alters the way they perceive risk [7,8], which is an inevitable factor in such decisions. To test our hypothesis, we conducted a study in which a state of power or a state of lack of power was experimentally induced. We then observed incentivized investment decisions after participants’ experiences of gain and loss.

### 1.1. Risky Financial Decisions in the Context of Gain and Loss

It is quite well documented that prior outcomes, both gains and losses, affect subsequent risky financial choices: [2,3,9]. However, the findings seem inconsistent about the specifics. On one hand, some studies have demonstrated that after losses, individuals make riskier decisions (the break-even effect [2]). For example, after experiencing a loss in a first gamble, participants tend to bet significantly more than they had initially planned in a second one [6]. On the other hand, there are studies demonstrating that more risk-seeking choices are made after a prior gain (the house-money effect [2]). For example, traders have been shown to take above-average risks in afternoon trading after morning gains [4], and another study found that priming memories of past wins caused people to become risk-seeking [5].

Numerous studies have attempted to explain these seemingly inconsistent findings. Weber and Zuchel [10] demonstrated that the effect of prior outcomes on risky choices depends on the format in which the decision problem is presented (i.e., whether a decision is depicted as a lottery task or an investment task). In this study, participants made two sequential financial decisions, and risk-taking after a loss was compared with risk-taking after a gain. The task was presented either as an investment portfolio decision or as a two-stage betting game. In the former condition, the risk taken by participants was greater following losses than following gains, whereas in the latter condition, the risk taken by participants was greater following gains than losses. Imas [11] demonstrated that different reactions to gains and losses in terms of the propensity to take risks can be explained by different reactions to realized versus paper losses. In this study, participants made four rounds of investment decisions. Some of the participants had their wealth positions realized after the third round, whereas the rest of the participants did not realize their earnings until the end of the experiment (a paper loss). The results showed that after realizing loss, individuals avoided risks, whereas if loss had not been realized, individuals were more risk-seeking. Another study showed that the nature of the previous task may constitute a factor that determines the direction of the relationship between the experience of gains and losses and risky financial decisions [9]. Its results showed that experiencing a gain in a financial task resulted in a higher propensity to take investment risks, while experiencing a loss made people less prone to take investment risks. The effects observed for gains and losses tasks were opposite: the experience of gain resulted in lower propensity to take investment risk and a non-financial loss resulted in higher propensity towards investment risk in subsequent tasks.

Gender is another factor that has been demonstrated to differentiate an individual’s propensity to take risk in response to gains and losses. For example, in a group of leisure life-time casino gamblers, males were more likely to bet more after a win, while females were more likely to bet more after a loss [12].

Emotion also plays a role in the effects of prior outcomes on risk decisions. One study comparing investment decisions of patients with stable focal lesions in brain regions related to emotion, patients with lesions that were unrelated to emotions, and participants without lesions demonstrated that the first group was less affected by the outcome of their decisions (both gains and losses) and consequently earned more money from their investment than participants from the other groups [13].

Taken together, it is not entirely clear why some people take greater financial risks after experiencing losses while others become more risk-seeking after experiencing gains. We propose another variable that might shed light on this issue: the power that one holds may influence one’s acceptable level of risk after an experience of loss/gain.

### 1.2. Power

Power is often defined as an asymmetry in control over valued resources (see [14] for a detailed discussion). This definition implies that those with high levels of power have relatively more control over resources (e.g., time, money, grades), while those with low power have relatively less control over those resources. Moreover, the latter depends on the former to obtain those resources [15]. There are various sources of power. According to Galbraith, power might rely on threats or negative sanctions (condign power), inducement (compensatory power), or persuasion (conditioned power) [16,17]. In his seminal book, Galbraith describes the instruments by which power is exercised. As an example of condign power, he lists various punishments, including physical and social ones—for example, the ability to destroy somebody’s standing in the community with a verbal attack. He clarifies the notion of compensatory power as buying submission, as in the case of submitting to a boss in return for wages or to a lobby in return for a bribe. Conditioned power is when people are persuaded to accept the will of others in the belief that it is right, virtuous, or proper—for example, when a politician makes a speech and persuades listeners to submit to his leadership.

Societies tend to be structured in social hierarchies in which certain people and groups achieve greater social status and power than others [18]. Consequently, individuals often find themselves either in positions of high power or in positions of low power—a manager vs. employees, a person with substantial resources vs. people with few resources, a professor vs. students. However, in some circumstances, the roles can be reversed: a boss can lack power when an employee leaves for a better job. Thus, a given person’s power can vary from situation to situation [19]. Accordingly, a great deal of research has demonstrated that power can be considered as a psychological state. Feelings of power or powerlessness can be activated by a number of factors (see [20] for a review), for example, structural factors (e.g., assigning people to the hierarchical roles of a boss or employee for the duration of a task) or cognitive factors (e.g., asking people to write about a time when they had/did not have power, or asking them to unscramble sentences containing words related to having or lacking power). Thus, it is possible to manipulate the subjective sense of power a person feels at a given time.

It is well documented that cues related to the possession of power create a sense of power, which in turn has a plethora of consequences for the way people process information, make decisions, and take actions (e.g., [21,22,23] for a review, see: [24]). A sense of power can affect a person’s decisions in many spheres, and financial matters are no exception. It has been demonstrated that powerful people are less likely than those with less power to spend all their money on current consumption [25] and demonstrate reduced temporal discounting [26]. Moreover, high power leads to a focus on the functional value offered by products and rejection of conspicuous consumption, while low power leads to a preference for high-status objects and conspicuous consumption [27].

### 1.3. Power and Financial Risk-Taking

In the context of risky financial decisions made after experiencing gain or loss, it is important to note that power increases the propensity to take risks. This has been demonstrated across a variety of situations, such as negotiations [21,28], taking a card in a game of blackjack [23], engaging in unprotected sex [21], marital infidelity [29], and risky food consumption [30]. The elevated propensity of powerful people to take risks has also been demonstrated in the case of financial decisions, both on corporate and individual levels. Specifically, power held by CEOs is positively related to excessive and unmanaged risk-taking by a firm; for example, misconduct of banks [31], the decision to pursue a strategy of specializing in subprime lending, which poses a high risk of default [32], or risk taken by banks [33]. Individual risky financial decisions are also related to the power possessed by the decision-maker. Increasingly high levels of power measured with the Sense of Power Scale [34] have been shown to be associated with the creation of riskier portfolios and more risky choices in the context of gambling. Similarly, a situationally induced high-power condition has been shown to result in greater investment and gambling risks than a low power condition [35]. However, these are the only studies on power and individual risky financial decisions, as far as we are aware; therefore, further studies that replicate and extend the effect are needed.

The positive effect of power on risk-taking is often explained by changes in the way decision-makers perceive their choices. Firstly, power impacts the way people perceive potential rewards and punishments. Research has demonstrated that high-powered individuals are more sensitive to potential gains and less sensitive to threats [22,36,37,38]. Power makes failure seem less probable and painful, and thus decreases the level of perceived risk of action [23,39,40] Furthermore, power changes the way the risk is perceived—it increases optimism [21,41], self-esteem [42], overconfidence [41,43], and perception of control over outcomes, even if they are beyond one’s reach [44].

### 1.4. Power and Decisions in the Context of Gains and Losses

When people make financial decisions, they usually have some previous experience of gains and losses. Hence, they make their financial choices in a specific decision context—sometimes it is their first decision ever, but more often they have a gain or loss fresh in their memory. Sometimes they might make their risky financial decisions having in mind an even longer history of successes and failures (e.g., a gain preceded by a loss, or a loss preceded by a previous gain). 

Although, to the best of our knowledge, the effect of power on risky decisions made after an experience of gain or loss has not been explored before, there are reasons to assume that people with power and powerless people will differ in their willingness to accept risk after such events. This hypothesis is based on the observation that people with high and low power perceive gains and losses differently. Specifically, a study by Lammers and Burgmer [45] indicated that, compared to those without power, powerful people tend to selectively attribute success internally and failures externally (i.e., showing a stronger self-serving tendency). 

Furthermore, research on the effects of power on an individual’s self-perception, especially self-esteem and overconfidence, supports the assumption that people in a position of power will differ in their reactions to failure and success from people with no power. Firstly, increased power leads to increases in global self-esteem, while decreases in power reduce self-esteem [42,46]. At the same time, one of the major differences between high and low self-esteem individuals is the way they react to failures [7,8]. The former seem to respond in a way that counteracts the potential negative impact of such experiences by focusing on their strengths and positive feelings about themselves [7,47]. Conversely, after a failure, individuals with low self-esteem focus on their weaknesses and shortcomings [7,47]. Secondly, power leads to overconfidence in the accuracy of one’s knowledge, thoughts, and beliefs, even in conditions when accuracy is in their financial self-interest [41,48,49] Importantly, studies show that overconfidence tends to persist despite performance feedback [50,51,52]; therefore, information about a single financial loss is not likely to reduce it.

Based on these studies, there are reasons to believe that being in states of power and lack of power affect financial decisions, including risky ones, after experiencing gains and losses. However, to the best of our knowledge, this issue has not been investigated before. Our follow-up analyses will also examine decisions made after a gain preceded by a loss and after a loss preceded by a gain.

### 1.5. Hypotheses & Study Overview

Considering the aforementioned gaps in the research literature, we conducted an incentivized study investigating the research questions and hypotheses described below. 

Firstly, taking into consideration the scarcity of studies demonstrating the impact of power on risky financial decisions and the value of replication, we aimed to verify previous findings that power enhances willingness to take risks when making investment decisions [35]. Therefore, we hypothesized that:

**Hypothesis** **1** **(H1).** *The state of having power increases the propensity to take investment risks while the state of lacking power decreases this propensity.*

Secondly, based on the literature and the reasoning presented in the previous paragraph, we formulated two research questions concerning the impact of power on investment choices made in the context of gains and losses.

**Research Question 1(RQ1):** Do states of having and lacking power alter the propensity to take investment risks in a decision context of previous gains and losses?
**Research Question 2 (RQ2):** Do states of having and lacking power alter the intensity of reactions to gains and losses?

Finally, it is important to note that previous studies on power focused mainly on only one source of power, compensatory power, which relies on incentives. However, power might also stem from other sources—for example, it might rely on negative sanctions and threats [16]. We assume that the previously observed relationships between power and risk are independent of the source of power. This would indicate that this relationship is universal, but this has not been demonstrated. Hence, we formulated the following hypothesis:

**Hypothesis** **2** **(H2).** *Relationships between power and propensity to take investment risks will be observed for both condign and compensatory power.*

In this study, based on the definition of power explored above, we operationalized the state of having power as a situation in which one has control over resources valued by others and the state of lacking power as a situation in which an individual does not have control over resources, but someone else does control them and regulates this access. Moreover, we took into account two sources of power distinguished by Galbraith: condign power and compensatory power. In our study, the first source of power refers to the possibility to decide whether to reward another person, and the second to whether to withhold additional remuneration.

## 2. Materials and Methods

### 2.1. Aim of the Study

The study aimed to examine the roles of power and lack of power in explaining investment choices made in changing decision contexts of gains and losses.

### 2.2. Method

#### 2.2.1. Participants

Polish working adults (*N* = 645; 329 female and 316 male; age 18–70 years, *M* = 41.47 years, *SD* = 13.47) took part in the study. The study was conducted using the online Polish ARIADNA participant panel, which has over 110,000 active adult panel members. E-mail invitations were sent to potential participants diversified in terms of their age, gender, and level of education. Respondents were awarded points for participating, which they could later exchange for rewards from a pool of several hundred products offered by the platform running the panel. Additionally, extra points were awarded to participants depending on their performance on an investment task. Informed consent was obtained from all participants.

To establish appropriate sample sizes, a priori power analysis was conducted using G*Power [53]. This showed that, given α = 0.05 and 0.80 power, a sample size of 582 participants would be sufficient to detect small effects (ƒ = 0.1) in our planned analyses. Our final sample was 10% larger (the panel delivered a bigger sample than we had ordered), allowing us to detect a slightly smaller effect (ƒ = 0.09).

#### 2.2.2. Materials and Procedure

**States of having (or lacking) power: experimental manipulation**. States of having power (condign power, compensatory power), states of lacking power (condign power, compensatory power), and a neutral state (in terms of power) were induced with five adapted scenarios successfully used in previous studies [35]. States of having power were induced by putting participants in a position that allowed them to evaluate and reward (compensatory power) or punish (condign power) other people for their performance. States of lacking power were induced by putting participants in the position of being the subject of an evaluation that was related to the possibility of being either rewarded (lack of compensatory power) or punished (lack of condign power) by another person—see Supplementary Materials for a description of the pilot study procedure and statistical analyses.

*Having-power conditions*: At the beginning of the procedure, participants in both having-power groups were informed that other panelists belonging to the same research panel had been given a creative task the previous week. The creative task involved writing three intelligible sentences in which they had to use three given words in such a way that it was difficult to guess which words had been provided. Next, they were asked to evaluate the performance of one of the panelists on this task. Participants in the compensatory-power condition decided whether to award this panelist with extra points, whereas participants in the condign-power condition decided whether to punish this panelist by depriving them of extra points.

*Lacking-power conditions:* At the beginning of the procedure, participants in the lacking-power group were informed that they would be asked to perform a creative task at the end of the study and that another panelist would be asked to evaluate their performance. Participants in the lacking-compensatory-power group learned that this panelist would decide whether to reward them with extra points, and participants in the lacking-condign-power group learned that this participant would punish them by depriving them of extra points. Next, they were presented with the same three sentences that participants from the having-power group evaluated (ostensibly, so that they could understand the task better). At the end of the procedure, these participants were asked to write their own three sentences. 

*Control group:* The procedure in the control group was similar to the procedure for the lacking-power groups, but they did not receive any information about the evaluation of their work. 

The full text of the experimental instructions is provided in the Supplementary Materials.

**Investment choices in a changing decision context task**—this task aimed to measure the riskiness of investment choices made in a varied decision context (initial decision, decision after gain, decision after loss, decision after gain preceded by loss, and decision after loss preceded by gain). It consists of two parts (variants) with three investment choices in each. At the beginning of each part, participants were provided with instructions. They were informed that they would make three decisions about a sum of 10,000 PLN (~USD 2400) each. During each decision, they would split this amount between an interest-free bank account and investments in stocks by declaring how much money they would like to invest. They were also informed that, after each decision, they would receive feedback about changes in the stock price and their current balance (calculated after taking into account changes in the stock price). They also learned that this task would be incentivized and the extra points earned in the study would depend on the participant’s final balance after all investment decisions. 

Next, a chart showing changes in the price of a hypothetical stock over the course of 12 months was presented. Participants were informed that the chart and its shape were computer generated. Next, the participants were asked to make their first decision. Then, they were informed about the change in stock price and their current balance. The same procedure was applied for the second and third decisions. Finally, at the end of each part of the investment choices task, participants were informed about their total balance and the number of extra points they would be awarded. If a participant decided to assign all the money to the interest-free bank account in all the decisions, they would receive four extra points. However, if the participant decided to invest in stocks, they could receive between one and seven extra points.

*Variant 1*: gain preceded by a loss. In this variant of the tool, after Decision 1 (initial choice), participants were informed that the stock price dropped. Next, they made Decision 2 (choice after having initially experienced a loss) and were informed that the stock price rose. Finally, they made Decision 3 (choice after a gain preceded by a loss) and were informed that the stock price had not changed. 

*Variant 2*: loss preceded by a gain. In the second variant of the tool, after Decision 1 (initial choice), participants were informed that the stock price had risen. Then they made Decision 2 (choice after having initially experienced a gain) and were informed that the stock price had dropped. Finally, they made Decision 3 (choice after a loss preceded by a gain) and were informed that the stock price had not changed. 

*Indicators*: The amount of money assigned to stocks in each decision was taken as an indicator of investment riskiness. This task enabled us to observe the level of each participant’s investment riskiness in the following contexts: (1) initial decision, (2) decision after a gain, (3) decision after a loss, (4) decision after a gain preceded by a loss, and (5) decision after loss preceded by gain. Moreover, the differences in the amount of money assigned to stocks between Decisions 1 and 2, as well as between Decisions 2 and 3, in each variant of the task were treated as indicators of the intensity of reaction to a change in the decision context. 

**Procedure.** At the beginning of the study, participants were randomly assigned to one of the five experimental conditions—either a state of power (compensatory or condign), lack of power (compensatory or condign), or the control condition—and were subsequently subjected to the experimental manipulation. Next, participants completed both variants of the investment choice task separated by an unrelated task. The additional unrelated task was introduced to convince participants that both parts of the investment choice task, although similar, were independent. To control for any undesired order effects, the variants of the investment choice task were presented to the participants in random order. after this, participants in the lacking-power and control groups were asked to perform the creative task. At the end of the data collection phase of the study, all participants were reminded about the outcome of the incentivized investment choice task and paid according to their performance.

## 3. Results

### 3.1. Investment Riskiness across Decision Contexts and Levels of Power

To check whether the experimental groups differed in investment riskiness in particular decision contexts, two 5 (experience of having power: having-condign-power, having-compensatory-power, lacking-condign-power, lacking-compensatory-power, control—between subjects IV) by 3 (decision context: initial choice, Decision 2, Decision 3) mixed-design ANOVAs were conducted, with the amount of money assigned to stocks by participants as a dependent variable, one for each variant of the investment choice task. Descriptive statistics for each variable are presented in Table 1.

#### 3.1.1. Investment Riskiness across Decision Contexts and Power Status—Variant 1: A Gain Preceded by a Loss

The mixed-design ANOVA showed a significant effect of decision context (*F*[2,1173] = 94.17, *p* < 0.001, *η*^2^ = 0.13). People decided to assign significantly more money to stocks in their initial decision than after having first experienced a loss (*t*(644) = 10.28, *p* < 0.001), and significantly less money than after a gain preceded by a loss (*t*(644) = −3.70, *p* < 0.001). Moreover, participants assigned less money to stocks after having first experienced a loss than after a gain preceded by a loss (*t*(644) = −12.81 *p* < 0.001).

A significant effect of having power was also observed (*F*[4,640] = 39.87, *p* < 0.001, *η*^2^ = 0.20). Participants who had power (both condign and compensatory) assigned more money to stocks than those who had no power (both condign and compensatory), and more than the control group (all *p*s < 0.001). Moreover, participants who experienced a state of lacking power (both condign and compensatory) assigned less money to stocks than did the control group (both *p*s < 0.001).

A significant interaction between the experience of having power and decision context was obtained (*F*[7,1173] = 19.79, *p* < 0.001, *η*^2^ = 0.11). Further ANOVA analysis showed that there were significant differences between the groups in terms of their propensity to invest in stocks in all analyzed decision contexts (Decision 1/Initial decision—*F*[4,640] = 10.70, *p* < 0.001; Decision 2/After loss—*F*[4,640] = 33.11, *p* < 0.001; Decision 3/Gain after loss—*F*[4,640] = 67.92, *p* < 0.001).

Further *t*-tests revealed that in the case of every decision, both having-power groups were prone to invest significantly more money in stocks than the control group and both lacking-power groups. Moreover, in the case of every decision, participants who experienced a state of lacking power (both types) assigned less money to stocks than the control group. There were no significant differences observed in the amount of money assigned to stocks between the having-condign-power and having-compensatory-power groups, nor between the lacking-condign-power and lacking-compensatory-power groups in any decision made in the task. Related statistics are given in the Supplementary Materials.

#### 3.1.2. Investment Riskiness across Decision Contexts and Power Status—Variant 2: A Loss Preceded by a Gain

The mixed-design ANOVA showed a significant effect of decision context (*F*[2,1225] = 27.72, *p* < 0.001, *η*^2^= 0.04). Participants invested more in Decision 2 (after gain) than in Decision 1 (initial decision, *t*(644) = 6.13, *p* < 0.001) and more than in Decision 3 (loss after gain, *t*(644) = 7.26, *p* < 0.001). The difference between the amount of money assigned to stocks in the initial choice and Decision 3 was nonsignificant (*t*(644) = 0.91, *p* = 0.36)

A significant effect of having power was observed (*F*[4,640] = 38.51, *p* < 0.001, *η*^2^ = 0.19). Both having-power experimental groups assigned more money to stocks than the control group and both lacking-power groups (all *p*s < 0.001). Moreover, the lacking-power groups (compensatory and condign) invested less than the control group (both *p*s < 0.001). There were no differences in money assigned to stocks observed between both having-power groups (*p* = 0.99) nor between the two lacking-power groups (*p* = 0.99).

However, a significant interaction between the experience of having power and decision context was observed (*F*[8,1225] = 6.17, *p* < 0.001, *η*^2^ = 0.04). Further ANOVA analysis showed that there were significant differences between the groups in terms of their propensity to invest in stocks in all analyzed decision contexts (Decision 1/Initial decision—*F*[4,640] = 15.93, *p* < 0.001; Decision 2/After gain—*F*[4,640] = 25.51, *p* < 0.001; Decision 3/Loss after gain—*F*[4,640] = 46.29, *p* < 0.001).

Further *t*-tests revealed that, in each decision, participants who experienced having power (both types) were prone to invest significantly more money in stocks than participants from the control group and participants who lacked power (both types). Moreover, in each decision, participants who lacked power (both types) assigned less money to stocks than did the control group. No significant differences in terms of the amount of money assigned to stocks in the case of each decision were observed between the having-condign-power and having-compensatory-power groups, nor between the lacking-condign-power and lacking-compensatory-power groups. Related statistics are given in the Supplementary Materials.

### 3.2. Intensity of Reaction to Changes in Decision Context and Levels of Power

To better understand the obtained results, we conducted two additional mixed-design ANOVAs to check whether there were differences between experimental groups in terms of the intensity of reactions to the changes in the decision context. Both ANOVAs were conducted using a 5 (experience of having power: having-condign-power, having-compensatory-power, lacking-condign-power, lacking-compensatory-power, control—between subjects IV) by 2 (change in decision context: between Decisions 1 and 2, between Decisions 2 and 3) schema, with the difference in money assigned to stocks between consecutive decisions as a dependent variable.

#### 3.2.1. The Intensity of Reaction to Changes in Decision Context and Power Status—Variant 1: A Gain Preceded by a Loss

The mixed-design ANOVA showed a significant effect of change in decision context (*F*[1,640] = 219.85, *p* < 0.001, *η*^2^ = 0.26). In general, the intensity of the reaction to a loss (being the first experience) was smaller than to a gain preceded by a loss (*t*(644) = 14.89, *p* < 0.001).

In addition, the effect of having power was significant (*F*[4,640] = 31.90, *p* < 0.001, *η*^2^= 0.17), with the two having-power groups being less sensitive to context changes than both lacking-power groups (all *p*s < 0.001) and the control group (*p*_compensatory_ = 0.004; *p*_condign_ < 0.001), and both lacking-power groups being more sensitive to context changes than the control group (*p*_compensatory_ < 0.001; *p*_condign_ =0.003).

There was no significant interaction effect between change in decision context and power status (*F*[4,640] = 0.286, *p* < 0.887, *η*^2^ < 0.01). However, one-way ANOVAs showed significant differences between the experimental groups in the intensity of their reaction to loss, both as initial experience (*F*[4,640] = 12.50, *p* < 0.001) and as gain after loss (*F*[4,640] = 10.53, *p* < 0.001). Further *t*-test analyses showed that the reaction to loss as initial experience was smaller among the having-power groups (both condign and compensatory) than among those from the lacking-power groups and the control group. Moreover, the intensity of reaction to gain after loss was greater among participants in both having-power groups than among participants from the two lacking-power experimental groups and the control group. Sensitivity to initial loss among the lacking-power groups was greater than in the control group, while the reaction to gain after loss was greater among the control group than both lacking-power groups. No differences in reaction to initial loss and reaction to gain after loss between the two having-power experimental groups and between the two lacking-power experimental groups were observed. Related statistics are given in the Supplementary Materials.

#### 3.2.2. The Intensity of Reaction to Changes in Decision Context and Power Status—Variant 2: A Loss Preceded by a Gain

No significant effect of change in decision context was observed in the mixed-design ANOVA (*F*[1,640] = 0.62, *p* = 0.43, *η*^2^ = 0.001). However, a significant effect of experimental condition was observed (*F*[4,640] = 4.03, *p =* 0.003, *η*^2^ = 0.03). In general, participants in the having-compensatory-power group were less sensitive to decision context changes than were the control group (*p* = 0.03) and the lacking-condign-power group (*p* = 0.001). The other differences between the analyzed groups were not significant.

Moreover, a significant interaction effect between change in decision context and having power was observed (*F*[4,640] = 12.58, *p* < 0.001, *η*^2^ < 0.07). Further one-way ANOVAs showed significant differences between the experimental groups in their reaction to initial gain (*F*[4,640] = 3.76, *p* = 0.005) and to loss after gain (*F*[4,640] = 4.59, *p* < 0.001). Reaction to initial gain was greater among participants in both having-power groups than in both lacking-power groups and the control group. No significant differences were observed between the two lacking-power groups, between the having-power groups, or between each of the lacking-power groups and the control group.

In terms of reaction to a loss after a gain, both lacking-power experimental groups reacted to a greater extent than did each of the having-power groups and the control group. The differences between the lacking-power groups, between the having-power groups, and between both having-power groups and the control group were not significant. Related statistics are given in the Supplementary Materials.

## 4. Discussion

The present study demonstrated that powerful people and those lacking power differ in their willingness to take financial risks and respond to gains and losses differently, both in terms of the amount of money they are willing to invest after such experiences and in terms of the intensity of reaction that is evoked by prior outcomes. Moreover, we observed no differences between people having compensatory and condign power in terms of their investment decisions.

The conclusion that people possessing power, people lacking power, and controls differ when it comes to the level of investment risk they are willing to take is based on a comparison of investment decisions made by these three groups after experiencing gains and losses. The present study shows that, in general, powerful people are prone to invest more money in stocks than the other groups and individuals lacking power tend to invest less money than the others. Importantly, this was observed regardless of prior experience—initially, after gain, after loss, and after experiencing gain after loss and loss after gain. These findings are in line with previous studies demonstrating that powerful people are more willing to take financial risks (e.g., [35]), as well as risks in a variety of other domains (see Introduction). However, they not only replicate previous results, but also substantially extend our understanding of the impact of power on risk-taking by demonstrating that this result is robust and can be observed in a variety of decision contexts. Most importantly, however, this result adds to a vast body of research on decisions made after prior gains and losses. Previous studies have found inconsistent patterns of reactions to prior outcomes. Here, we demonstrate that power could be one of the factors that explain these seemingly contradictory results. We observed that, after gains, people high in power were more willing to take risks than controls, and people lacking power were less willing to take risks than controls. Similar patterns of results were observed in the case of decisions made after losses—powerful people took more investment risks than controls and people lacking power took less risks than controls. This indicates that people from these two groups react differently to prior gains and losses, which is consistent with the reasoning used to formulate our hypotheses.

The conclusion that people high and low in power differ in terms of the intensity of their reactions to gains and losses is based on a comparison of the amount of money invested in stocks in two consecutive decisions. These two decisions were made in different contexts (initial, after gain, after loss, after gain preceded by loss, after loss preceded by gain). Hence, we were able to observe how changes in decision context impacted the intensity of reactions. The first finding concerns the way powerful people react to gains. The results indicate that their reaction is greater than that of controls and people lacking power. Moreover, such a relationship was observed both in the case of an initial gain and when a gain was preceded by a loss. The second finding concerns the way powerful people react to losses. This reaction was smaller than that of controls and people lacking power, but only when an initial loss was experienced. When a loss was preceded by gain, there was no difference between the powerful and the controls, but there was still a significant difference between these groups and individuals lacking power. It seems that the experience of previous gain acts as a buffer for both powerful and power-neutral people that makes them behave more similarly in terms of risky investment choices after loss. The next finding concerns how people lacking power react to losses. This reaction was greater than in controls, both when an initial loss was experienced and when a loss was preceded by a gain. Finally, we also observed that powerless people showed a smaller reaction to gains than did the control group, but only when the gain was preceded by a loss. No differences were observed in the case of an initial gain. It seems that for people who lack power, the experience of loss is more meaningful than the experience of gain, and previous loss may even make them less sensitive to subsequent gains compared to people in a power-neutral state. Generally, our findings provide evidence that powerful people are more driven by gains than by losses, while those lacking power are more sensitive to losses compared to gains. This relationship seems to be strong enough that, among powerful individuals, a prior gain might neutralize a subsequent experience of loss, whereas among powerless individuals, a prior loss might neutralize the experience of subsequent gains.

The study presented in this article provides one more finding. The results show that the type of power (compensatory vs. condign) did not differentiate people’s risky investment choices, either for people in a position of power or for people lacking power. This might indicate that the role of power is universal, regardless of its source. However, given that Galbraith distinguished three types of power—compensatory, condign, and conditioned [16]—it would be interesting to conduct additional studies to examine whether the relationships observed in the present study also occur in situations of induced conditioned power.

Despite our study’s interesting findings, there are some limitations. Specifically, although the study was incentivized and participants were paid based on their performance, their payoff was not comparable to real-life gains and losses on the stock market. Taking into account that hypothetical rewards allow the prediction of actual behavior [54,55], we are confident in the obtained results. However, a study that would allow us to observe real-life individual risky financial decisions and their relationships with the decisionmaker’s power would be of great value.

The present study has several theoretical implications. The results further our understanding of decisions made by individual investors by identifying another situational factor—the power held by a decision-maker—that might impact the level of risk they are willing to take, and which might alter one’s responses after experiencing gains and losses. Thus, our results contribute to the research on risk-taking, power, and choices made in response to prior outcomes

The results of this study also have practical implications. First, each variable identified as important in predicting and understanding people’s personal financial choices is valuable, given recent economic shocks associated with the 2008 banking crisis, the global economic crisis that may well result from the COVID-19 pandemic, and the growing problem of indebtedness. Second, establishing factors that may illuminate our understanding of the mechanisms underlying risky financial choices is essential for promoting good financial decision making. Revealing such mechanisms to interested decision-makers might enable them to gain insight into their decision processes and help improve their decision making. Awareness of relevant situational factors, even those that might be transient and seemingly unrelated to subsequent decisions, might alter a person’s propensity to take risks and make consumers more sensitive and cautious when making risky choices. As such, the present results are of interest to all institutions that deal with risk decisions.

## 5. Conclusions

This study demonstrated that the power held by a decision-maker impacts their responses to gains and losses when making risky financial choices, both in terms of the amount of money assigned to investments and in terms of the reaction evoked by prior outcomes. This implies that the role of situational factors in risky financial decision making is substantial. Not only can the consequences of previous choices impact subsequent ones, but one’s power status at the moment of deciding is also crucial, as it changes the way one reacts to such consequences.

## Figures and Tables

**Table 1:** Descriptive statistics in the total sample and experimental groups.

	Total	Compensatory Power	Condign Power	Control	Lacking Compensatory Power	Lacking Condign Power
First variant of investment choice task (gain preceded by a loss)						
Decision 1 (initial decision)	4164.48 (2885.06)	5015.05 (2854.02)	4946.62 (3047.44)	4128.68 (2895.85)	3375.94 (2590.25)	3357.68 (2586.27)
Decision 2 (after initial loss)	3451.32 (2887.35)	4821.28 (2837.35)	4754.12 (3176.30)	3451.94 (2577.55)	2108.57 (2211.81)	2123.22 (2239.91)
Decision 3 (after gain preceded by loss)	4519.81 (3136.49)	6313.05 (2860.59)	6579.99 (2882.56)	4435.89 (2883.88)	2621.78 (2240.38)	2640.64 (2215.96)
Second variant of investment choice task (loss preceded by a gain)						
Decision 1 (initial decision)	4972.80 (2854.91)	5876.96 (2463.97)	5886.13 (2564.00)	5152.71 (2849.67)	3930.74 (2694.37)	4019.92 (3052.43)
Decision 2 (after initial gain)	5567.36 (3074.35)	6918.27 (2640.75)	6907.67 (2805.68)	5218.65 (2944.24)	4360.61 (2847.86)	4434.21 (3066.46)
Decision 3 (after loss preceded by gain)	4868.76 (3189.08)	6567.71 (2678.57)	6579.56 (2757.94)	4788.88 (3123.41)	3091.43 (2750.90)	3323.02 (2751.27)

Note: The table shows *M* values with *SD*s in parentheses. All values are in PLN.

## Data Availability

Complete data for all studies and the original materials used to conduct this research can be found on the Open Science Framework (OSF) website: https://osf.io/v7ye8/?view_only=d5640bf3c23d4421bd4328cae3c79a1e (access on 23 October 2021).

## Supplementary Materials

The following are available online at https://www.mdpi.com/article/10.3390/ijerph182312834/s1: the pilot study for the experimental manipulation procedure, experimental manipulation instructions, simple contrasts of experimental conditions in riskiness of investment choices, simple contrasts of experimental conditions in intensity of reaction to decision context changes, descriptive statistics of intensity of reaction to decision context change in the total sample and experimental groups.
