# Supplementary material for: How Power Influences Decision-Makers’ Investment Behavior in the Domains of Loss and Gain
Source: Int J Environ Res Public Health. 2021 Dec 6;18(23):12834. doi: 10.3390/ijerph182312834 (PMC8657608; doi:10.3390/ijerph182312834)
Supplement: Supplementary file 1 [file ijerph-18-12834-s001.zip › ijerph-1454000-supplementary.pdf]

How power influences decision-makers' investment behaviour in the domains of loss and gain

Sekścińska, K.; Rudzinska-Wojciechowska, J.

SUPPLEMENTARY MATERIALS

1. The pilot study for the experimental manipulation procedure

The pilot study was conducted as manipulation check; the experimental procedure aimed to induce state of power (condign or compensatory) or lack of power (condign or compensatory) and included control conditions.

To test the experimental procedure, a two-wave pilot study was conducted. Data for the first wave of the study were collected as the first part of a larger online survey, with the later parts of the survey unrelated. Because of this, it was not possible to tailor the sample size to the exclusive demands of the pilot study. The second wave of the study was conducted after one week break.

Participants

In total, 509 participants took part in the first wave of pilot study, 291 women (57.2%) and 218 men (42.8%), aged 18 to 79 years (*M* = 41.39, *SD* = 14.46). However, only 415 people completed both waves of the study, 233 women (56.1%) and 182 men (43.9%), aged 18 to 79 years (*M* = 42.11, *SD* = 14.33).

Materials and procedure

In the first wave of the study, participants were randomly assigned to one of the experimental conditions (state of having compensatory power, n = 99; state of having condign power, n = 107; state of lacking compensatory power, n = 95; state of lacking condign power, n = 102; control, n = 106).

States of having power (condign power, compensatory power), states of lacking power (condign power, compensatory power) and neutral (in terms of power) were induced with five scenarios prepared specifically for the studies that would be conducted using an online research panel of registered participants

States of having power were induced by putting participants in a position that allowed them to evaluate and reward (compensatory power) or punish (condign power) other people for their performance. States of lacking power were induced by putting participants in a position of being the subject of an evaluation that was related to possibility of being either rewarded (lack of compensatory power) or punished (lack of condign power) by another person.

State of having power conditions: At the beginning of the procedure, participants in both powerful groups were informed that other panelists belonging to the same research panel had been given a creative task the previous week. The creative task involved participants writing three sensible sentences in which they had to use three provided words in such a way that it was difficult to guess which word had been provided. Next, they were asked to evaluate a performance of one of the panelists in this task. Participants in compensatory power condition decided whether to award this panelist with extra points whereas participants in compensatory power condition decided whether to punish this panelist by depriving the panelist of extra points.

State of lacking power conditions: At the beginning of the procedure, participants in the lack of power group were informed that they would be asked to perform a creative task at the end of the study, and that another panelist would be asked to evaluate their performance. Participants in a lack of compensatory power group learnt that this panelist would decide whether to reward them with extra points and participants in lack of condign power group learnt that this participant would punish them by depriving them of extra points. Next, they were presented with the same three sentences that participants from the powerful group evaluated (ostensibly, so that they could understand the task better). At the end of the procedure, these participants were asked to write their own three sentences.

Control group: The procedure in the control group was similar to the procedure for lack of power groups participants but did not receive any information about evaluation of their work.

After completing the experimental tasks, all the participants were asked two manipulation check questions related to the experimental situation (in a rotated order):

Q1: *How would you rate your power in this task?* (scale: 1- I had no power at all [someone else had it] to 10 - I definitely had power [someone else didn't]; or answer: this task did not evoke any associations with having or not having power).

Q2: *How would you rate the control you had in this task over allocating valuable resources (points in this case)*? (scale: 1-I had absolutely no control [someone else had it] to 10 - I definitely had control [someone else didn't]; or the answer: this task did not evoke any associations with having or not having control)

Then the participants completed the Generalized Sense of Power Scale (Anderson and Galinsky, 2006) on which participants are asked to report their generalized beliefs about the power they have in their relationships with others. Participants were asked to rate their agreement with eight statements such as "In my relationships with others I can get others to do what I want" on a scale from 1 ("strongly disagree") to 5 ("strongly agree"). Four items were reverse coded, and then responses were averaged to create an indicator of each participant's sense of power.

One week after the first wave of the study, the participants were invited to the second wave of the study, in which they again completed the Generalized Sense of Power Scale (Anderson and Galinsky, 2006) to measure their general sense of power unrelated to the experimental manipulation.

Results

We analyzed the frequencies of "this task did not evoke any associations with having or not having power" and "this task did not evoke any associations with having or not having control" answers in all the analyzed groups of participants. In accordance with our expectations, in case of almost all participants from four experimental groups the experimental study evoked associations with power and 98% of participants from the control group reported that the task did not evoke any associations with power (Table 1).

Table S1

Frequencies of answers indicating lack of associations with power during the experimental tasks in the experimental groups.

	Having compensatory power group[1]	Having condign power group[2]	Lacking compensatory power group[3]	Lacking condign power group[4]	Control group[5]
"This task did not evoke any associations with having or not having power"	3 (3%)	3 (2.8%)	3 (3.2%)	2 (1.9%)	98 (96.1%)
"This task did not evoke any associations with having or not having control"	0 (0%)	3 (2.8%)	3 (3.2%)	3 (2.8%)	98 (96.1%)

[1] *n* = 99; [2] *n* = 107; [3] *n* = 95; [4] *n* = 102; [5] *n* = 106

Next, we analyzed the differences between the experimental groups in their answers to two manipulation check questions. We excluded from the analyses those who reported that the task did not evoke any associations with power. In accordance with our expectations, participants from each of the having power experimental groups rated the power they had in the task higher than those from each of the lacking power experimental group. Such differences were observed for both manipulation check questions. No differences in answers toQ1 and Q2 were observed between two having power experimental groups as well as between the two lacking power experimental groups (Table 2 and Table 3).

Table S2.

Frequencies and descriptive statistics for perceived power.

	Q1	Q2
Having compensatory power group	*n* = 96, *M* = 8.86, *SD* = 1.40	*n* = 99, *M* = 8.65, *SD* = 1.61
Having condign power group	*n* = 104, *M* = 8.49, *SD* = 1.95	*n* = 104, *M* = 8.52, *SD* = 1.96
Lacking compensatory power group	*n* = 92, *M* = 5.27, *SD* = 2.80	*n* = 92, *M* = 5.45, *SD* = 2.73
Lacking condign power group	*n* = 104, *M* = 5.83, *SD* = 2.27	*n* = 103, *M* = 5.13, *SD* = 2.32

Table S3.

Differences in perceived power during experimental task between experimental conditions.

	Q1	Q2
Having compensatory power group vs Lacking compensatory power group	$t(132) = 11.068, p < .001$	$t(145) = 9.791, p < .001$
Having compensatory power group vs Lacking condign power group	$t(173) = 11.492, p < .001$	$t(182) = 12.578, p < .001$
Having compensatory power group vs Having condign power group	$t(187) = 1.569, p = .118$	$t(197) = 0.507, p = .613$
Having condign power group vs Lacking compensatory power group	$t(160) = 9.228, p < .001$	$t(163) = 8.955, p < .001$
Having condign power group vs Lacking condign power group	$t(201) = 9.074, p < .001$	$t(199) = 11.357, p < .001$
Lacking compensatory power group vs Lacking condign power group	$t(175) = 1.513, p = .132$	$t(180) = 0.876, p = .382$

Further analyses aimed to check if the manipulation task changed participants' general sense of power. We compared the results of all the five research groups. The results showed significant differences between the analyzed groups ($F(4,504) = 10.328$, $p < .001$).

Further t-test analyses showed that people from having compensatory power group (*M* = 27.21, *SD* = 5.57) had higher general sense of power than people from both lacking power groups ($M_{compensatory}$ = 23.73, $SD_{compensatory}$ = 5.86; $M_{condign}$ = 23.38, $SD_{condign}$ = 6.37), and than the control group (*M* = 25.22, *SD* = 4.41). Moreover, people from having condign power group (*M* = 26.83, *SD* = 4.99) had higher general sense of power than people from t, and than the control group. There were no significant differences observed between the two having power groups as well as between two lacking power groups (Table 4).

Table S4.

Differences between groups in general sense of power after experimental manipulation.

	t
Having compensatory power group vs Lacking compensatory power group	$t(190) = 4.242$, $p < .001$
Having compensatory power group vs Lacking condign power group	$t(202) = 4.596$, $p < .001$
Having compensatory power group vs Control group	$t(186) = 2.813$, $p = .005$
Having compensatory power group vs Having condign power group	$t(197) = 0.515$, $p = .607$
Having condign power group vs Lacking compensatory power group	$t(186) = 4.026$, $p < .001$
Having condign power group vs Lacking condign power group	$t(199) = 4.402$, $p < .001$
Having condign power group vs Control group	$t(206) = 2.484$, $p = .014$
Lacking compensatory power group vs Control group	$t(174) = 2.004$, $p = .047$
Lacking condign power group vs Control group	$t(187) = 2.428$, $p = .016$
Lacking compensatory power group vs	$t(199) = 0.404$, $p = .686$

Finally, we checked if there was a difference between the analyzed groups in their general sense of power level, when measured one week after the manipulation, to be sure that the observed effects are not biased. We analyzed the data from the second wave of the study. There were no significant differences observed between analyzed groups ($F(4,410) = 0.662$, $p = .619$, Thus, the differences observed in SOP level in the first wave of the study did not result from the natural discrepancies between the groups.

2. Experimental manipulation instruction

Experimental conditions: having compensatory and condign power (fragments of the instruction specific for each of the having power group are in italic with the text for having condign power group presented in brackets).

Last week, research concerning creativity was conducted among our panelists. Participants were asked to write short stories comprising three sensible sentences in which they had to use three provided words in such a way that it was difficult to guess which word had been provided. In other words, the words provided should have been concealed as much as possible. *The best stories will be used in future studies and rewarded with a cash prize, converted into additional points for use in the Ariadna Panel store (The worst stories will not be used in future studies and their authors will be deprived of a cash prize, converted into additional points for use in the Ariadna Panel store).*

Today your task is to evaluate the stories and to decide *whether to award their authors with extra points and if so – how many points should be awarded* (*whether their authors should be deprived of the prize and if so – how much should it be reduced*). You will also decide whether the story *will be used in further studies* (*will be excluded from use in further research*).

Read the story below and answer the questions provided.

In the story the following three words had been concealed: plane, pencil case, rope (either plural or singular).

Story. 1. (author number 217)

This September Krystian is going to a new school, which makes him very excited, and motivates him to prepare everything in advance. He has a new backpack with a picture of a plane on it and a colorful pencil case full of colorful felt tip pens and crayons. Krystian can't wait for the first day of school because jump rope contest will take place and Krystian is strong and thinks that he will win.

1) *Evaluate the task performance (very poor/excellent)*

2) *Should this story be used in further research*? (*Should this story be excluded from use in further research?)* (yes/no)

3) *The author of the story may receive an extra prize from the organizers of the research. The prize is in Polish zloty and the money will be converted to points exchangeable for rewards. Considering the quality of the story, should the author of this story be awarded? Using the scale provided below, indicate the amount of the prize to be awarded to the author of this story or choose 0 if in your opinion the author should not receive a reward. (PLN 0 – PLN 10) (The maximum amount of the prize that author of the story may receive from the organizers of the research is PLN 10, which will be converted to points exchangeable for rewards. Considering the quality of the story, should the author be deprived of part or whole of the prize ? Using the scale provided below, indicate how much would you reduce the prize for the author of this story or choose 0 if in your opinion the authors prize should not be reduced at all. (PLN 0 – PLN 10))*

Experimental conditions: lacking compensatory power and lacking condign power (fragments of the instruction specific for each of the having power group are italic with the text for having condign power group present in brackets).

Your task is to write a short story comprising three sensible sentences using three provided words in such a way that it is difficult to guess which word has been provided. In other words, the words provided should be concealed as much as possible. *The best stories will be used in future studies and rewarded with the money that will be converted to points exchangeable for rewards offered by the platform running the panel. Another panelist will evaluate your story and decide whether you will be awarded extra points and if so – how many points should be awarded.*

(For a story you create, you can receive a financial reward of up to PLN 10, that will be converted to points exchangeable for rewards offered by the platform running the panel, and your story may be used in a future studies. Another panelist will evaluate your story and decide whether you should be deprived of some or all of the prize (the panelist may reduce the maximum amount of PLN 10 arbitrary, even to PLN 0).

This person will read your story and answer the following questions:
1) Evaluate the task performance (very poor/excellent)
2) *Should this story be used in further research*? (*Should this story be excluded from use in further research?)* (yes/no)
3) *The author of the story may receive an extra prize from the organizers of the research. The prize is in Polish zloty and the money will be converted to points exchangeable for rewards. Considering the quality of the story, should the author of this story be awarded? Using the scale provided below, indicate the amount of the prize to be awarded to the author of this story or choose 0 if in your opinion the author should not receive a reward. (PLN 0 – PLN 10) (The maximum amount of the prize that author of the story may receive from the organizers of the research is PLN 10, which will be converted to points exchangeable for rewards. Considering the quality of the story, should the author be deprived of some or all of the prize ? Using the scale provided below, indicate how much would you reduce the prize for the author of this story or choose 0 if in your opinion the authors prize should not be reduced at all. (PLN 0 – PLN 10))*

To understand better this task, you will see the story written by other panelists.
In the story the following three words had been concealed: plane, pencil case, rope (either plural or singular).
Story. 1. (author number 217)

This September Krystian is going to a new school, which makes him very excited, and motivates him to prepare everything in advance. He has a new backpack with a picture of a plane on it and a colorful pencil case full of colorful felt tip pens and crayons. Krystian can't wait for the first day of school because jump rope contest will take place and Krystian is strong and thinks that he will win.

Now write your own story using the following three words: lamp, penguin, tree (singular or plural)

Control condition

Your task is to write a short story comprising three sensible sentences using three provided words in such a way that it is difficult to guess which word has been provided. In other words, the words provided should be concealed as much as possible.

To understand better this task, you will see the story written by other panelists. In the story the following three words had been concealed: plane, pencil case, rope (either plural or singular).

Story. 1. (author number 217)

This September Krystian is going to a new school, which makes him very excited, and motivates him to prepare everything in advance. He has a new backpack with a picture of a plane on it and a colorful pencil case full of colorful felt tip pens and crayons. Krystian can't wait for the first day of school because jump rope contest will take place and Krystian is strong and thinks that he will win.

Now write your own story using the following three words: lamp, penguin, tree (singular or plural)

3. Simple contrasts of experimental conditions in riskiness of investment choices

Table S5.

Simple contrasts of experimental conditions in riskiness of investment choices (Variant 1 of Investment choice task).

		t	*df*	*p*
Decision 1 (Initial decision)	Condign power vs No condign power	4.70	640	<.001
	Condign power vs Control	2.52	640	.01
	Condign power vs Compensatory power	0.20	640	.85
	Condign power vs No compensatory power	4.68	640	<.001
	No condign power vs Control	-2.17	640	.03
	No condign power vs Compensatory power	-4.57	640	<.001
	No condign power vs No compensatory power	0.05	640	.96
	Control vs Condign power	-2.36	640	.02
	Control vs no Condign power	2.19	640	.03
	Condign power vs No condign power	4.55	640	<.001
Decision 2 (After loss as a first experience)	Condign power vs No condign power	8.53	234	<.001
	Condign power vs Control	4.02	248	<.001
	Condign power vs Compensatory power	0.18	255	.86
	Condign power vs No compensatory power	8.35	235	<.001
	No condign power vs Control	-4.52	252	<.001
	No condign power vs Compensatory power	-7.88	236	<.001
	No condign power vs No compensatory power	-0.05	255	.96
	Control vs Condign power	-3.65	252	<.001
	Control vs no Condign power	4.39	249	<.001
	Condign power vs No condign power	7.73	238	<.001

Decision 3 (After gain preceded by loss)	Condign power vs No condign power	11.49	235	<.001
	Condign power vs Control	5.21	252	<.001
	Condign power vs Compensatory power	-0.75	255	.46
	Condign power vs No compensatory power	11.35	233	<.001
	No condign power vs Control	-5.67	241	<.001
	No condign power vs Compensatory power	-12.50	249	<.001
	No condign power vs No compensatory power	-0.07	255	.95
	Control vs Condign power	-6.02	260	<.001
	Control vs no Condign power	5.57	240	<.001
	Condign power vs No condign power	12.35	246	<.001
	Control vs no Condign power	4.68	244	<.001
	Condign power vs No condign power	11.56	255	<.001

Table S6.

Simple contrasts of experimental conditions in riskiness of investment choices (Variant 2 of Investment choice task).

		t	*df*	*p*
Decision 1 (Initial decision)	Condign power vs No condign power	6.06	256	<.001
	Condign power vs Control	2.17	249	.03
	Condign power vs Compensatory power	-0.03	256	.98
	Condign power vs No compensatory power	5.29	237	<.001
	No condign power vs Control	-3.56	258	<.001
	No condign power vs Compensatory power	-6.06	263	<.001
	No condign power vs No compensatory power	-0.25	247	.80
	Control vs Condign power	-2.19	255	.03

	Control vs no Condign power	3.06	249	.002
	Condign power vs No condign power	5.30	243	<.001
Decision 2 (After loss as a first experience)	Condign power vs No condign power	7.17	640	<.001
	Condign power vs Control	4.73	640	<.001
	Condign power vs Compensatory power	0.03	640	.98
	Condign power vs No compensatory power	6.86	640	<.001
	No condign power vs Control	-2.42	640	.02
	No condign power vs Compensatory power	-7.25	640	<.001
	No condign power vs No compensatory power	-0.21	640	.84
	Control vs Condign power	-4.77	640	<.001
	Control vs no Condign power	2.18	640	.03
	Condign power vs No condign power	6.93	640	<.001
Decision 3 (After gain preceded by loss)	Condign power vs No condign power	9.91	640	<.001
	Condign power vs Control	5.03	640	<.001
	Condign power vs Compensatory power	-0.03	640	.97
	Condign power vs No compensatory power	9.11	640	<.001
	No condign power vs Control	-4.88	640	<.001
	No condign power vs Compensatory power	-10.10	640	<.001
	No condign power vs No compensatory power	-0.66	640	.51
	Control vs Condign power	-5.14	640	<.001
	Control vs no Condign power	4.15	640	<.001
	Condign power vs No condign power	9.28	640	<.001

4. Simple contrasts of experimental conditions in intensity of reaction to decision context changes

Table S7.

Simple contrasts of experimental conditions in intensity of reaction to decision context changes (Variant 1 of Investment choice task).

		t	*df*	*p*
Reaction to loss as a first experience	Condign power vs No condign power	-6.28	142	<.001
	Condign power vs Control	-2.24	134	.03
	Condign power vs Compensatory power	0.01	159	.99
	Condign power vs No compensatory power	-6.30	134	<.001
	No condign power vs Control	2.18	245	.03
	No condign power vs Compensatory power	5.46	219	<.001
	No condign power vs No compensatory power	0.14	256	.89
	Control vs Condign power	2.05	185	.04
	Control vs no Condign power	-2.08	237	.04
	Condign power vs No condign power	-5.43	212	<.001
Reaction to gain preceded by loss	Condign power vs No condign power	-4.79	240	<.001
	Condign power vs Control	-2.05	246	<.001
	Condign power vs Compensatory power	1.06	212	.86
	Condign power vs No compensatory power	-5.27	198	<.001
	No condign power vs Control	2.06	225	<.001
	No condign power vs Compensatory power	4.36	187	<.001
	No condign power vs No compensatory power	0.03	238	.96
	Control vs Condign power	2.53	234	<.001
	Control vs no Condign power	-2.21	185	<.001

	Condign power vs No condign power	-4.54	162	<.001

Table S8.

Simple contrasts of experimental conditions in intensity of reaction to decision context changes (Variant 2 of Investment choice task).

		t	*df*	*p*
Reaction to gain as a first experience	Condign power vs No condign power	-2.40	253	.02
	Condign power vs Control	-2.92	228	.004
	Condign power vs Compensatory power	-0.07	247	.95
	Condign power vs No compensatory power	-6.30	134	.02
	No condign power vs Control	-2.32	247	.29
	No condign power vs Compensatory power	-1.06	221	.04
	No condign power vs No compensatory power	2.03	243	.95
	Control vs Condign power	-0.06	250	.009
	Control vs no Condign power	2.62	255	.33
	Condign power vs No condign power	-5.43	212	<.05
Reaction to loss preceded by gain	Condign power vs No condign power	-3.84	241	<.001
	Condign power vs Control	-0.30	220	.76
	Condign power vs Compensatory power	0.09	231	.93
	Condign power vs No compensatory power	-2.41	184	.02
	No condign power vs Control	2.89	225	.004
	No condign power vs Compensatory power	3.32	262	.001

No condign power vs No compensatory power	0.46	221	.64
Control vs Condign power	0.34	259	.74
Control vs no Condign power	-1.91	235	.05
Condign power vs No condign power	-2.24	232	.03

5. Descriptive statistics of intensity of the reaction to the decision context change in a total sample and experimental groups

Table S9.

Descriptive statistics of intensity of the reaction to the decision context change in a total sample and experimental groups

		Having compensatory power group	Having condign power group	Lacking compensatory power group	Lacking condign power group	Control group
*Variant 1**	Intensity of the reaction to loss being the first experience	-193.77 (371.62)	-192.50 (1189.18)	-1267.34 (1934.04)	-1234.46 (1810.62)	-676.74 (2419.02)
*Variant 1**	Intensity of the reaction to gain preceded by loss	1491.77 (1789.10)	1825.87 (3146.86)	513.21 (1463.58)	517.42 (1033.13)	983.95 (2152.72)
*Variant 2**	Intensity of the reaction to gain being the first experience	1041.31 (2086.99)	1021.54 (2707.24)	429.87 (2003.76)	414.29 (2188.02)	93.07 (265.88)
*Variant 2**	Intensity of the reaction to loss preceded by gain	-350.56 (1603.39)	-328.10 (2401.13)	-1269.18 (2212.66)	-1111.18 (3149.16)	429.77 (2475.56)

Note: The table presents the *M* values with *SD* in parentheses. All the values are in PLN
*Variant of investment choices in varying decision context task
